# Preliminary Evaluation of Automated Speech Recognition Apps for the Hearing Impaired and Deaf

**DOI:** 10.3389/fdgth.2022.806076

**Published:** 2022-02-16

**Authors:** Leontien Pragt, Peter van Hengel, Dagmar Grob, Jan-Willem A. Wasmann

**Affiliations:** ^1^Department of Otorhinolaryngology, Donders Institute for Brain, Cognition and Behaviour, Radboud University Medical Center Nijmegen, Nijmegen, Netherlands; ^2^Pento Audiological Center Twente, Hengelo, Netherlands; ^3^Department of Medical Imaging, Radboud University Medical Center, Nijmegen, Netherlands

**Keywords:** automated speech audiometry, (automatic speech recognition), automated speech recognition, (ASR), evaluation metric, hearing impairment, speech-to-text, voice-to-text technology

## Abstract

**Objective:**

Automated speech recognition (ASR) systems have become increasingly sophisticated, accurate, and deployable on many digital devices, including on a smartphone. This pilot study aims to examine the speech recognition performance of ASR apps using audiological speech tests. In addition, we compare ASR speech recognition performance to normal hearing and hearing impaired listeners and evaluate if standard clinical audiological tests are a meaningful and quick measure of the performance of ASR apps.

**Methods:**

Four apps have been tested on a smartphone, respectively AVA, Earfy, Live Transcribe, and Speechy. The Dutch audiological speech tests performed were speech audiometry in quiet (Dutch CNC-test), Digits-in-Noise (DIN)-test with steady-state speech-shaped noise, sentences in quiet and in averaged long-term speech-shaped spectrum noise (Plomp-test). For comparison, the app's ability to transcribe a spoken dialogue (Dutch and English) was tested.

**Results:**

All apps scored at least 50% phonemes correct on the Dutch CNC-test for a conversational speech intensity level (65 dB SPL) and achieved 90–100% phoneme recognition at higher intensity levels. On the DIN-test, AVA and Live Transcribe had the lowest (best) signal-to-noise ratio +8 dB. The lowest signal-to-noise measured with the Plomp-test was +8 to 9 dB for Earfy (Android) and Live Transcribe (Android). Overall, the word error rate for the dialogue in English (19–34%) was lower (better) than for the Dutch dialogue (25–66%).

**Conclusion:**

The performance of the apps was limited on audiological tests that provide little linguistic context or use low signal to noise levels. For Dutch audiological speech tests in quiet, ASR apps performed similarly to a person with a moderate hearing loss. In noise, the ASR apps performed more poorly than most profoundly deaf people using a hearing aid or cochlear implant. Adding new performance metrics including the semantic difference as a function of SNR and reverberation time could help to monitor and further improve ASR performance.

## Introduction

Automated Speech Recognition (ASR) has become increasingly sophisticated and accurate as a result of advances in deep learning, cloud computing, and the availability of large training sets (1, 2). The software converts speech into text using artificial intelligence models that have been trained on vast collections of speech containing millions of words. ASR software is widely available on most digital devices, including smartphones, tablets, or laptops. It is primarily used for voice commands (e.g., hey Siri!), at the workplace to create transcripts, or in class for taking notes. Recently, ASR has become available in online meetings (e.g., Microsoft teams) and video recordings (e.g., Google's Youtube) to provide automated captions. Also, several ASR-based speech-to-text apps have been developed for the hearing impaired and deaf, providing live captioning of conversations (2, 3), showing the potential of automation and artificial intelligence for hearing healthcare (4, 5). Early in 2020, we were confronted in our clinic with questions from patients related to the use of ASR apps for daily communication. These questions were especially common among patients with severe to profound hearing loss who visited our outpatient clinic to assess if they were eligible for a Cochlear Implant. Also, patients who had experienced sudden deafness, but had not yet been fitted with hearing aids, made use of an ASR app during their appointments. There was no or little experimental information at the time about the performance and usability of the ASR apps for hearing impaired persons beyond what was shared by developers. Nor did we have clear criteria for which groups of patients we might suggest the ASR apps to.

### Background

Since 2017, several ASR systems have claimed speech recognition performance close to that of normally hearing humans (1, 2). The most common metric to express ASR performance, used to underpin these claims, is the word error rate (WER). WER is calculated by adding the number of missing, wrong, and inserted words and dividing this by the total number of words (6). A lower WER score means better performance. The performance of ASR will be best for speech similar to the speech on which it was trained (7). It is therefore important to understand for what specific task an ASR is designed and how it is evaluated. Typically ASRs are evaluated on well-studied large (>100 h) collections of speech, referred to as a corpus. The SwitchBoard corpus and CallHome corpus are well-known collections of conversational phone calls (8), whereas Librispeech is a corpus comprising speech from public domain audiobooks. The SwitchBoard corpus consists of conversations over the phone between strangers about a given topic (9). The CallHome corpus consists of more informal conversations between friends and family (8). None of these corpora are ideal for use in acoustically challenging environments. The SwitchBoard and CallHome were collected under low noise and low reverberation conditions (9), and a large portion of the Librispeech corpus has undergone noise removal and volume normalization (10).

In order to obtain estimates of human speech recognition performance that could be used for comparison with ASR, some researchers have determined the WER among professional transcribers of speech from the SwitchBoard and CallHome corpora. Saon et al. (1) estimated the lowest (best) achievable WER, 5.1% for SwitchBoard and 6.8% for CallHome, based on the best score taken from three professional speech transcribers after a quality check by a fourth speech transcriber. Xiong et al. (2) on the other hand, followed more realistic industry standard procedures, which are similar to how speech is processed by ASR. The reported WERs were 5.9% for SwitchBoard, and 11.3% for CallHome.

For some commonly-used ASR systems, WERs of 5.1% (Microsoft) and 5.5% (IBM) have been reported using the SwitchBoard corpus (11), which is close to the performance of normal hearing professionals reported above (1, 2). Benchmark results of widely used ASR systems tested on the same corpora are not available to our knowledge. Google reported a WER of 4.9%, but used a non-public corpus (11). Koenecke et al. (7) compared the performance of ASR systems from Amazon, Apple, Google, IBM, and Microsoft to transcribe structured interviews using two recent developed corpora (CORAAL and AAVE). However, transcribing a structured interview is a very different task than transcribing a conversation in real-time in acoustically challenging environments. More ecologically valid tasks are needed that take into account the effects of noise, reverberation, talker accent, and slang, for instance, to provide a realistic estimate of ASR performance when used for conversations in daily life under various acoustic conditions.

### ASR for Hearing Impaired Listeners

For people with hearing impairments, there are specific user needs to consider when developing ASR apps. For example, these listeners might use both speechreading (12) and text reading of the ASR transcript from a screen. Speechreading conveys important non-verbal cues and nuances not included in a transcript and may enhance speech-in-noise abilities (13). However, without careful design, reading a transcript may interfere with someone's speechreading ability. Speaker identification cues [e.g., by color coding each speaker a feature in AVA (14)] may also direct the reader to the face of an active talker. Other design ideas include the notification of critical environmental sounds [a feature incorporated in Live Transcribe (15)], feedback to the speaker of their intelligibility of the ASR, or feedback to the speaker by making the transcript readable from two sides (e.g., mirrored) so that both the speaker and the listener can check the results [incorporated in Earfy (16)].

The settings where an ASR is used may also differ between individuals with impaired or normal hearing. For example, the settings where people with hearing loss use ASR may be more often in a more homely atmosphere between family members that might use more colloquial language or slang. That situation may be similar to closed caption for video series. The most common complaint of people with hearing loss is the reduced speech perception in complex listening environments including cocktail parties, restaurants, in conversations with their doctor, and family gatherings (15, 16). Adverse acoustic conditions, including low signal-to-noise, make it difficult for normal hearing listeners to understand speech and make the speech incomprehensible for persons with mild to profound hearing loss (17, 18). Finally, the speed of translation to accommodate a fluent conversation and the user interface to make it practical for older users and digitally less proficient users are factors to consider.

A standardized task that fully captures the skills of humans to recognize speech does not yet exist, to our knowledge. Such a task would need to account for factors as background noise, reverberation, accent, and speech impairment. This is needed to verify claims that ASR speech recognition performance is close to humans (1, 2) and should be done using diverse training datasets (7).

### Objective

This pilot study aimed to examine the speech recognition performance of ASR apps using audiological speech tests. We normally administer clinical audiology tests in patients from normal hearing to profound hearing loss to assess speech recognition. We tested the hypothesis that our clinical tests might thus provide objective metrics for performance of ASR systems for people with hearing loss, helping us to determine what range of hearing losses could benefit from ASR apps. In addition, we compared ASR results to normal hearing and hearing impaired listeners and evaluated if standard clinical audiological tests provide a meaningful and quick measure of the performance of ASR apps.

## Methods

Four different apps on two smartphones, with various operating systems, were tested on their ability to transcribe speech. For this project, the iOS operating apps were tested using an iPhone 6, and for the Android operating apps, a Samsung A3 was used. Both smartphone devices are widely used. We decided to select inexpensive ASR apps (<$10) for a user-license since they would be most widely used by our patients while the cost for ASR apps is not reimbursed in the Netherlands. The four apps tested were Ava and Earfy that both run on iOS and Android, Speechy iOS only, and Live Transcribe Android only. The tested apps were chosen by searching on the Internet on November 18th, 2019, for the best-known speech recognition apps for the hearing impaired and deaf as well as good reviews on the different app-stores. Also, the apps needed to be suitable to convert English and Dutch speech into text.

**Figure 1:**
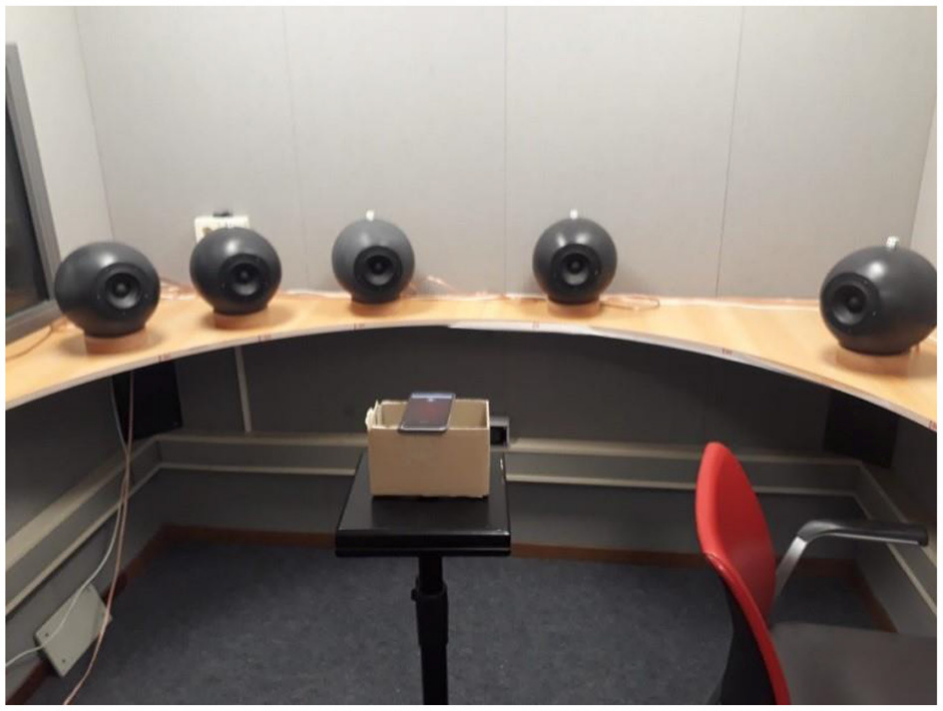
Set-up of the smartphone in front of the speaker.

The apps were evaluated in similar test conditions used to assess speech reception in human listeners in Dutch Audiology Centers according to best local clinical practice. The smartphones were placed one meter in front of a speaker in a sound treated room compliant with ISO 8253-1 (19). Standard clinical calibration protocols were used for all speech material. The microphone of the smartphone was aimed toward the speaker, which we assumed to be the optimal microphone orientation, at approximately the height of a listener's ears to resemble testing conditions when tested with human listeners (see Figure 1). The smartphone screen was facing upwards allowing the experimenter to read the text from the screen. Four different speech reception tests were performed to evaluate the app's ability to convert speech into text.

First, the apps were tested on speech recognition in quiet by converting a list of single words into text. The standard Dutch speech recognition test for this purpose is the Dutch CNC-test, which consists of phonetically balanced lists of twelve monosyllabic Dutch words in quiet [CNC-list, “Nederlandse Vereniging voor Audiologie;” (20)]. The words were played through a speaker, scored and displayed in a phoneme recognition score. All words consisted of three phonemes with a consonant-nucleus-consonant (CNC) structure. The first word was a test word and was not included in the scoring. A human observer performed the scoring by reading the word from the screen and counting the number of correct phonemes. Inserted phonemes were subtracted from the score according to the clinical scoring procedure (20). If a displayed word changed during the test, the final word was scored. A 100% phoneme recognition score was reached if all 33 phonemes of the 11 words were correct. Several lists were presented at an intensity level of 45, 55, 65, 75, and 85 dB sound pressure level (SPL) and the speech recognition as a function of presentation level (known in human listeners as speech audiogram) is plotted for each app. For comparison, normal hearing listeners achieve 100% phoneme recognition at 45 dB SPL (20).

Second, the Plomp-test (Dutch sentences in noise) was presented (21). The test consists of 13 sentences of 8 to 9 syllables presented in noise with the same averaged long-term spectrum as speech. A sentence was scored to be either correct, if the whole sentence was correctly presented on the screen, or incorrect, which was according to the conventional scoring procedure in clinical practice (22). The speech recognition threshold (SRT) in noise was defined as the signal-to-noise ratio (SNR) expressed in dB where on average 50% of the time the sentences were transcribed correctly, following the adaptive procedure described by Plomp and Mimpen (20, 21). The test was first performed without noise to obtain the SRT in quiet. Afterward, the masking noise level was set 15–20 dB above the SRT of the apps in the quiet situation, which was 70 dB SPL for all apps, to determine the speech reception threshold (SRT) in noise.

Third, a DIN-test (Digits-in-Noise) was performed. Digit triplets (e.g., 1 2 5) were presented in long-term average speech-spectrum noise via a 1-up, 1-down adaptive SNR procedure. SRT was expressed in dB SNR, where a listener can on average recognize 50% of the digit triplets correctly. A test series consisted of 24 triplets. The first four triples were not used to determine the test outcome. The noise level was set at a fixed level of 60 dB with an initial positive SNR of 6 dB. The stepsize to adjust the level of the triplets was 2 dB. The DIN-test has a measurement error in humans of 0.7 dB (23).

Fourth, a fragment of dialogue in Dutch and English at 72.2 dB(A) was presented through the speaker to recreate a more realistic listening setting. The Dutch dialogue was an introduction video of the Radboudumc with a female voice, talking clearly and at a normal pace (https://www.youtube.com/watch?v=zBJBD1-ePRw). For the English dialogue, part of an advanced English tutorial was played. In this video, a conversation could be heard between a male and female voice (https://www.youtube.com/watch?v=JtMgw2rCYSo&t=1s). The Dutch dialogue consisted of 256 words, while the English dialogue consisted of 248 words. After the whole dialogue was played, scoring was performed on the transcript outputted by the app. The number of missing, wrong, and inserted words was counted and expressed in the WER.

In the end, a test-retest was performed to provide insight into the accuracy of the apps. All apps were retested on the CNC-test. The test-retest reliability on the CNC-test was visually assessed using a Bland-Altman graph. The best scoring app on the DIN- and Plomp-test, one for iOS and one for Android, was retested for both speech-in-noise tests. The Root-Mean-Square-Difference (RMSD) was calculated for these results. No retest was performed for the dialogue.

## Results

**Figure 2:**
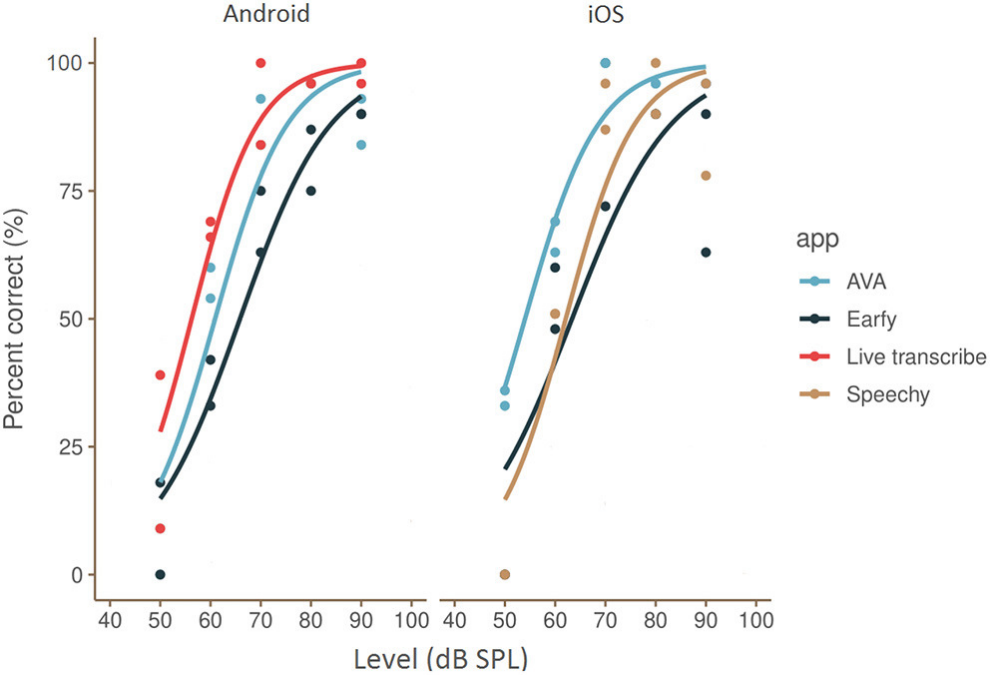
Speech recognition as a function of presentation level (in human listeners known as speech audiogram) of all ASR apps tested on an Android and iOS smartphone. The plotted lines are interpolated using a logistic function through the measured test-retest data-points. Left side, results of the Android apps, right side, results of the iOS apps.

The results for all apps on the Dutch CNC-test (words in quiet) are shown in Figure 2. Speech recognition as a function of presentation level was determined per app by interpolating a line using logistic regression on all available-data points (test and retest measurements). A 100% phoneme recognition was reached around 80 dB SPL for all apps except Earfy. Earfy (iOS and Android) scored 90% words correctly around 90 dB SPL. The shape of the app's “speech audiogram” curves look similar to the s-shaped psychometric curve of normal hearing listeners determined by Bronkhorst et al. (24) in 20 normal hearing university students. However, all app's SRT were between 50 and 60 dB SPL, which is 25 to 35 dB poorer than normal hearing listeners who have a SRT around 25 dB SPL (20).

**Figure 3:**
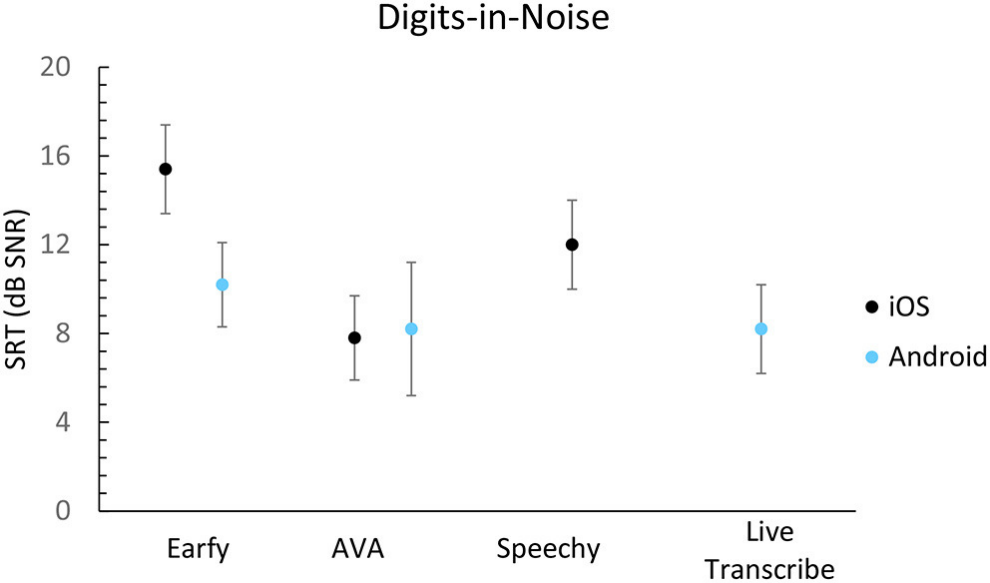
Digit in noise results expressed in SNR per app. A lower score is better. The error bars represent the standard deviation of the response of the app within a single list of triplets.

**Figure 4:**
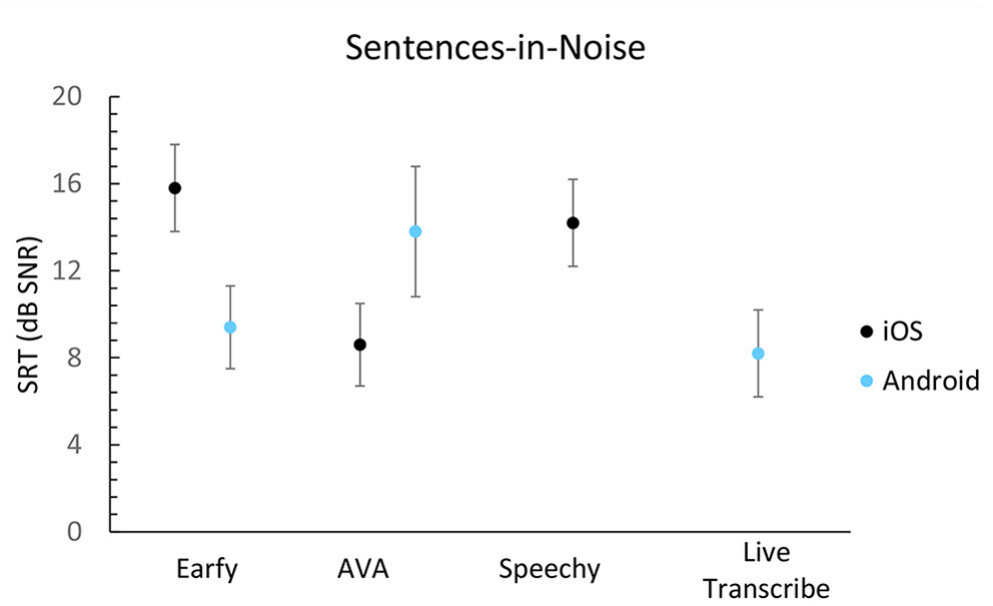
Sentences in noise results expressed in SNR per app. A lower score is better. The error bars represent the standard deviation of the response of the app within a single list of sentences.

The speech-in-noise results are shown in Figures 3, 4. All apps score a signal-to-noise ratio (SNR) higher than +8 dB on the DIN- and Plomp-test. Live transcribe (Android), and AVA (Android, iOS) achieved the best results on the DIN-test. Earfy on Android performed better than on iOS. Live Transcribe (Android) and AVA (iOS) achieved the best result using the Plomp-test. There was a notable difference between the operating systems for AVA and Earfy when measured with the Plomp-test.

**Figure 5:**
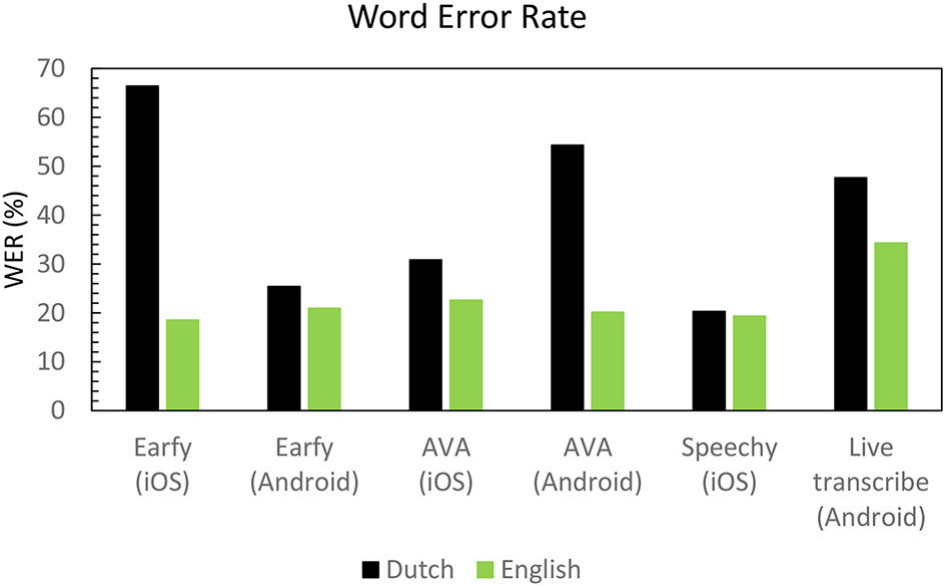
Word error rate in percentage of the dialogue in English and Dutch for the different apps.

In Figure 5, the WER scores for both the Dutch and English dialogue are shown. Overall, the dialogue in English (WER 19–34%) was more correctly converted into words than the Dutch (WER 25-66%) dialogue. Speechy (iOS) had best matching result for English and Dutch (WER of 19 and 20%). Earfy (iOS) showed the greatest difference between English and Dutch (WER of 19 and 66%).

**Figure 6:**
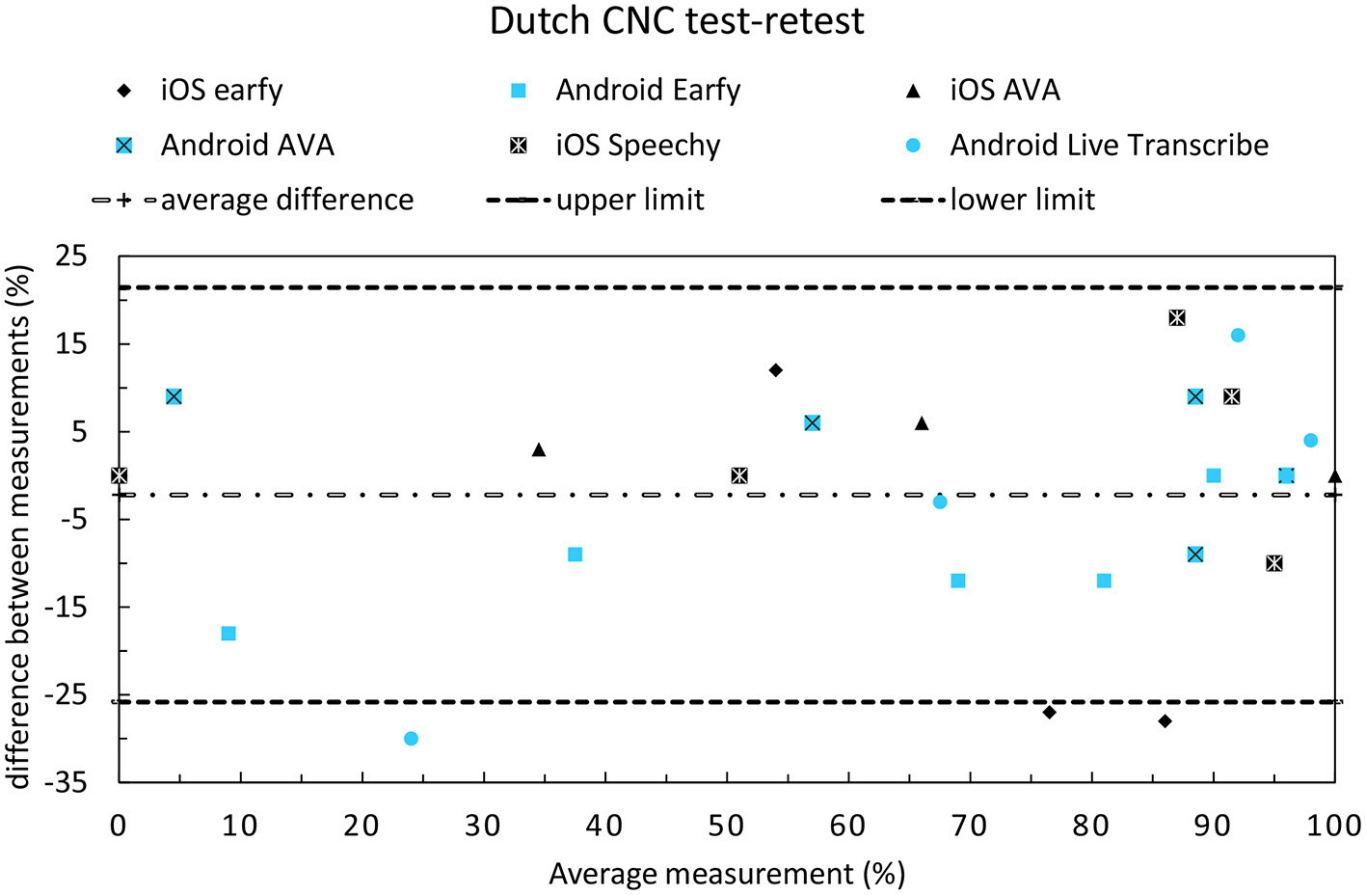
Bland-Altman plot to display the test-retest reliability of the CNC-test.

The test-retest reliability of the CNC-test can be seen in Figure 6. Visual inspection of the Bland-Altman plot for the CNC-test-test did not show signs of any systematic bias in the relationships between differences and averages. The test-retest comparison of the CNC-test showed three outliers. Earfy for iOS exhibited large differences between the measurements at 70 and 90 dB and Live transcribe (Android) had a large difference between measurements at 50 dB. The test-retest reliability on the DIN- and Plomp-tests was assessed for one Android and one iOS app. The test-retest difference expressed in RMSD on the DIN-test was 0.4 dB iOS Ava and 0.8 dB Android Live Transcribe, which we regard as acceptable since in normal hearing listeners tested monaurally using headphones, 90% of measurements are within 1.4 dB (measurement error is 0.70 dB) for a single list on the DIN-test (23). The RMSD on the Plomp-test was 0.6 dB iOS Ava and 2.0 dB for Android Live Transcribe.

## Discussion

### Main Results

None of the ASR apps achieved performance close to normal hearing listeners on audiological tests. In quiet, ASR apps performed similarly to listeners with a moderate hearing loss. When transcribing speech-in-noise, the ASR apps performed in the performance range of CI recipients. Sentences-in-noise provided a quick test to assess ASR performance since that test material provided more linguistic cues than digits-in-noise or lists of CNC words.

### Performance Compared to Human Listeners

The performance of the ASR apps on speech-in-quiet tests seems comparable to listeners with a moderate conductive hearing loss (30–35 dB threshold shift), which is known as disabling for certain activities in daily life (25). In comparison, Dingemanse and Goedegebure (26) found a mean score of 82% in 50 adult unilateral CI-recipients on the Dutch CNC-test tested in free field at 65 dB SPL, which is the level of conversational speech. This performance may be an overestimation for the average CI user since they excluded participants with a CNC-score below 60%. Kaandorp et al. (27) determined a mean score in free field at 65 dB SPL of 95% while using their preferred device in 24 hearing aid users with a moderate to severe hearing loss and 80% in 24 CI recipients. Only for speech at high-intensity levels, well above the level of conversational speech, do the apps achieve 90 to 100% speech reception. The poor performance at low speech intensity levels may be caused by hardware limitations, as discussed below in the section on hardware. The ASR may score lower due to the lack of contextual information provided in the test. The CNC-test was developed as an auditory test that requires little linguistic skill. The listener can only use the consonant-nucleus-consonant structure and the fact that the lists contain only familiar existing words. The alternative of using nonsense words, or nonsense sentences, would probably further deteriorate ASR performance while being a valid test for assessing auditory function with a lower effect of language skills by the subject (28). Most ASR are trained on sentences of realistic conversations (8). The strength of (deep learning) ASR is based on using contextual information from a natural language processing model (29). That contextual information is not available in word testing.

The performance of the ASR apps on the Digits-in-Noise test was very limited compared to humans. Normal hearing listeners achieve on the DIN-test, monaurally using headphones, an SNR of −8.8 dB (23). CI recipients rated on the same criteria as normal hearing listeners, typically achieve DIN scores ranging from +3 to−6 dB. For instance, Kaandorp et al. (27) found an average SNR of −1.8 (±2.7) dB in a group of 18 adult unilateral CI recipients in free field test conditions. The ASR is at a disadvantage because in the DIN-test, contextual information is lacking and the priors for the ASR and human are not the same. When doing a digits-in-noise test, a human will only report digits. For the ASR it is not a 10-class problem but a problem with several thousand alternatives. The apps tend to construct sentences rather than separate numbers. For conversations where it is important to catch a number, such as the price of an item, the DIN-test might be a useful measure.

The performance of ASR apps on sentences in noise (Plomp-test) was very limited and much poorer than in people with a moderate hearing loss (21). Normal hearing listeners have an SRT at an SNR of −8 to −10 dB (21), while the best ASR apps achieved +8 dB scores. Kaandorp et al. (27) found a mean SRT on Dutch Sentences in noise by scoring keywords of +2.1 dB for 24 hearing aid users (tested on their preferred ear) with moderate to severe hearing loss and +8.0 dB for 24 unilateral CI recipients. In CI-recipients, evaluation of speech-in-noise is often performed scoring keywords, instead of full sentences as used in the original procedure by Plomp and Mimpen (20, 21). In another study, Kaandorp et al. (30) found a significant difference of 1.0 dB in favor of a keyword scoring procedure compared to scoring full sentences. However, this 1.0 dB keyword effect does not account for the large difference between the app's performance and the performance of hearing aid users in noise. On the Plomp-test, which provides more linguistic information than the CNC- and DIN-test, the app's performance is far below that of the majority of hearing impaired listeners and similar to the range of outcomes in CI-recipients.

Sentences with and without noise (Plomp-test) could be considered as a performance metric for ASR apps in difficult listening conditions. Possibly with more natural sentences to provide even more linguistic cues. Testing through a loudspeaker has the advantage that it takes the effect of room acoustics into account, making the test condition more realistic. Instead of a sound booth, a room with more representative acoustics for daily situations (e.g., the reverberation time of a classroom or using babble noise instead of speech-shaped noise) would provide even more representative results. The current scoring procedure of the Plomp-test, based on fully correct sentences, leads to very high SNRs that may underestimate the practical value of ASR for hearing impaired persons. For instance, if an ASR in a conversation under noisy conditions provides keywords, it may already benefit the person with hearing impairment. One could easily adopt the Plomp-test by determining the WER score on a fixed SNR level to simulate above example. Or alternatively, accept a higher number of mistakes (compared to none) in the adaptive test by using keywords (30). Besides audiological test outcomes, the systematically collected feedback by groups of users (e.g., a focus group) would be very helpful to further improve the accessibility and usability of ASR apps for hearing impaired listeners.

In longer dialogues, all tested apps provided a running English transcript with a WER around 19–34%. This roughly corresponds to 60–80% correct word (~1-WER) scores and this is in the same range as for persons with profound hearing loss who use a cochlear implant (31) and better than hearing aid users with a profound hearing loss (32). For these groups, the use of the ASR apps tested here would likely provide benefits.

### Hardware and Platform Variability

A possible explanation for the poor performance at low levels could be the smartphone's microphone gain settings and limited dynamic range rendering soft sounds undetectable (33). We chose a microphone orientation, directing it to the speaker that we assumed was optimal for the task. However, we did not check the directionality of the built-in microphones. In actual use, the microphone orientation could be suboptimal, for instance, if a listener positions the device such that it enables better reading of the transcript from the screen. Also in group settings, the user will likely put the device flat on a table and thus not always point the microphone to the talker. We did not investigate the effect of suboptimal microphone orientation. Another explanation for the level dependence in quiet could be pre-processing. Most ASR systems usually normalize the input (34). Potentially the ASR systems classify soft sounds as non-speech or not of interest.

In English, there is not much difference between the apps or between the operating platforms. Therefore, we do not expect differences in the Dutch version to stem from hardware differences between the smartphones (e.g., microphone sensitivity) but from the implementation of the Dutch language in the specific app or the used training data. The difference between iOS and Android was only visible in Dutch. In Dutch, Earfy (iOS) and Ava (iOS) score significantly poorer.

There was no consistent difference favoring either iOS or Android versions of the apps. Earfy performed better on Android, while AVA performed better on iOS. For prospective users, the performance of the app depends on language, and may depend on the platform.

### Limitations

The administered tests did not include the effect of accents or speech impairments [e.g., deaf speech; (7, 35)]. The displayed transcripts changed during the dialogue, and the transcript was evaluated at the end of the dialogue instead of in real-time. When reading the transcript in real-time, the performance of the speech recognition apps might be better or worse due to the changing words in real-time to construct a logical sentence.

When measuring performance in noise, an adaptive SNR procedure was used. The effect of noise could be more extensively studied by evaluating ASR by determining the Word Recognition Score (the convention in the field of audiology) or the Word Error Rate (the convention in the field of ASR research) on several fixed SNR levels (e.g.,−5, 0, +5 and +10 dB SNR) that correspond to realistic listening conditions for people using a hearing aid (36). For ecological valid measures, the effect of different fluctuating noise maskers should be considered (37, 38). Babble-noise or traffic noise is much more realistic than (artificial) steady-state speech-shaped noise. In the end, the performance of the ASR must be robust enough that users will put their trust in these apps even in formal situations such as a conversation with their doctor or audiologist.

In this study, only (audiological) speech-to-text performance of the apps was measured. The usability, processing speed, effect on speechreading, and readability of the transcript were not evaluated. Other researchers looked into requirements for speed and user interface and concluded that those are important factors to improve usability (39). We expect that an increasing number of ASR apps will adhere to accessibility guidelines to improve usability for the elderly and people with disabilities as promoted by the Web Accessibility Initiative (40).

The number of apps tested in this study is limited. We did not perform a standardized procedures for literature review (e.g., PRISMA) to find and include ASR apps for this pilot study. In English, more apps may be available than in Dutch and we did not include expensive state-of-the-art (professional) ASR systems.

Other factors to consider not included in this pilot study are the distance between speaker and listener, especially in these times of social distancing and the effect of face masks on a speaker's voice and intelligibility (41). Feedback about voice quality could help the speaker adopt a more intelligible speaker style. The errors made by the ASR may be complementary or redundant to the errors made by persons with hearing loss. We did not study the error patterns. A potential way to determine the complementary effect of ASR could be to evaluate speech-recognition in noise using an audiovisual presentation mode, instead of the audio-only mode that was used in this study, in three distinct aided conditions. (1) participants with hearing loss aided with hearing aid or CI. (2) participants with hearing loss aided with hearing aid or CI and using an ASR app, (3) performance by the ASR app only. Studying the difference between these conditions reveals the added benefit and may penalize ASR systems not designed for simultaneous speechreading and text reading.

### Metrics to Evaluate Personalized ASR Performance

Instead of the quick audiological tests we performed here, a more conventional and elaborate evaluation method would be to record several hours of conversations with hearing impaired users (including realistic lexicon and acoustics) via a smartphone while the screen is oriented such that the user can read the transcript. Subsequently, one could create transcripts of the recordings by human transcribers as ground truth, pass the recordings through several ASR apps and determine a performance rating based on WER and other automated metrics such as the semantic distance between the ASR transcript and ground truth (42).

ASR may benefit from domain-specific evaluation tools and have domain-specific applications. For instance, Miner et al. (43) developed a metric based on symptom-focused language in psychotherapy. A domain-specific, or even person-specific factor is that prelingually deaf people often have a speech impairment, leading to lower comprehensibility both for normal hearing listeners who are not accustomed to deaf speech and for ASR apps that are not specifically trained on deaf speech. Fortunately, generic ASR models can be used as a pre-trained model that subsequently is trained on a particular task including a-typical speech, accents, or acoustic conditions without incurring the cost of training a full model (44). Recently, researchers from Google started a project, called Parrotron, to create personalized models which could better convert deaf speech than generic ASR systems. WER dropped from 89.2% for the generic ASR to 32.7% for the finetuned ASR for a single prelingually deaf subject (35). In addition, the Parrotron system can synthesize the speech of a speech impaired person (i.e., voice conversion) to make the speech sound more natural and comprehensible to the untrained ear.

Metrics as, for example, the WER (SNR, RT), or semantic difference (SNR, RT), as functions of signal-to-noise ratio and reverberation time (RT) can provide more ecologically valid estimates of the benefits ASR apps could provide in daily life. Representative SNR values could include −5, +10, +30 (quiet) dB SNR. For ecological valid measures, realistic fluctuating noise maskers should be used (37, 38). Reverberation times typically encountered in daily life to consider are 0, 0.5, and 2.5 s, which corresponds to ideal, classroom (45), and church (46) room acoustics. Presenting the ASR performance using the WER (SNR, RT) reduces the need to study the characteristic of the corpus on which the ASR was trained and or evaluated.

### Future Benefits for Audiologists

ASR apps can provide benefits in conversations between patients and their audiologists (47). In addition, ASR technology, when further developed, can play a role in computational approaches to audiology (4). For instance, if personalized ASR apps further develop so that atypical speech is better captured, and if ASR achieves normal hearing performance on audiology tests it may provide another use case: patients could perform self-testing (i.e., automated speech audiometry) by repeating the speech they hear to an ASR system trained on their particular voice replacing or enhancing the task of the professional in the audiology center (48). Manual calculation of complex evaluation metrics is not suitable in clinical settings given the excessive time required and may lead to inter-rater variability (49). Automated speech audiometry using algorithms to score performance can be a valuable complement to automated pure-tone threshold audiometry (50). For example, Venail et al. (48) validated a semi-automatic speech procedure using customized word-lists, in part provided by the subject to include familiar words. The customized word-lists were recorded with the subject's own voice to incorporate personalized acoustic and articulatory parameters. Speech recognition was evaluated on the customized word-list using an algorithm to determine automatically the number of correctly repeated phonemes. In addition, the use of ASR could open venues to improved (automated) scoring methods in audiology tests. Ratnanather et al. (51) demonstrated how one can automate the alignment of phonemes based on the minimum edit distance between the source speech and the utterances of the subject in real time. Visualizing this alignment may provide insights to clinicians about what phonological errors are made.

A factor of variability in rating procedures is that in many speech-in-noise tests, the test is made easier for CI recipients by only scoring correct keywords rather than full sentences (28, 30). Although scoring keywords makes the test accessible to a larger population, it reduces the discriminative power between higher- and lower-educated native listeners (30). An ASR could facilitate an automated scoring procedure that differentiates between errors. For instance, using semantic difference between the ASR transcript and ground truth, errors that lead to semantically similar sentences are weighted favorably, leading to a better outcome metric in terms of how well hearing impaired persons can participate in a conversation under adverse circumstances.

## Conclusion

None of the ASR apps achieved performance close to normal hearing listeners on audiological tests. No app stood out from the others on performance level. On audiological speech tests in quiet, ASR apps performed similarly to listeners with a moderate hearing loss. When transcribing speech-in-noise, the ASR apps performed in the performance range of CI recipients. Sentences-in-noise provided a quick test to assess ASR performance. Additional performance measures are needed to evaluate ASR apps. Besides the speech material, also type of noise and the presentation mode audio-only vs. audiovisual need to be considered. Adding new performance metrics including the semantic difference as a function of SNR and reverberation time can help to monitor and further improve ASR performance. Clinicians can use benchmarks based on such metrics to counsel prospective users and may benefit from automated procedures. Several hearing impaired listeners, especially CI recipients, report that they benefit from the apps in certain situations (47), which is in accordance with the results of converting a dialogue into text and may stem from complementary error patterns of ASR not investigated here. Personalized ASR could increase the number of listeners enjoying the benefits of ASR.

## Data Availability Statement

The raw data supporting the conclusions of this article will be made available by the authors, without undue reservation.

## Author Contributions

LP, J-WW, and PH conceptualized the study. LP, PH, and DG collected the data. LP and J-WW took the lead in drafting the manuscript. All authors contributed to the data interpretation, reviewed the results, and edited the manuscript.

## Conflict of Interest

The authors declare that the research was conducted in the absence of any commercial or financial relationships that could be construed as a potential conflict of interest.

## Publisher's Note

All claims expressed in this article are solely those of the authors and do not necessarily represent those of their affiliated organizations, or those of the publisher, the editors and the reviewers. Any product that may be evaluated in this article, or claim that may be made by its manufacturer, is not guaranteed or endorsed by the publisher.
